# Nanomaterial-based strategies for oral biofilm management: functionalized implants and targeted delivery systems

**DOI:** 10.3389/froh.2026.1789632

**Published:** 2026-04-08

**Authors:** Liu Yuming, Xu Zhenkun, Sun Yixin, Chen Xi, Ding Jingzhe, Peng Yan, Yu Wenjun, Chen Hui, Jiang Wentao

**Affiliations:** 1 Faculty of Dentistry, Universiti Kebangsaan Malaysia, Kuala Lumpur, Malaysia; 2 Center for Health Ageing and Wellness, Faculty of Health Sciences, Universiti Kebangsaan Malaysia, Kuala Lumpur, Malaysia; 3 Smile Dental Clinic, Tianjin, China; 4 Affiliated Hospital of North China University of Science and Technology, Tangshan, Hebei, China; 5 School of Stomatology, Shandong Second Medical University, Affiliated Hospital of Shandong Second Medical University, Weifang, China

**Keywords:** delivery systems, dental implants, nanomaterials, oral biofilm, peri-implantitis

## Abstract

Biofilm-associated infections, particularly peri-implantitis, threaten the long-term success of dental implants, and conventional debridement or chemotherapy often fails against mature biofilms. Nanomaterials offer multifunctional strategies to control infection while supporting osseointegration. This mini-review (2015–2025) summarizes nanomaterial-based approaches for managing implant-associated oral biofilms, including passive surface functionalization, active delivery systems, externally triggered therapies, and host-directed osteoimmunomodulation. Their potential should be interpreted in light of evidence maturity, safety—especially for ROS-based modalities—and long-term tribological and manufacturing limitations. We also highlight practical selection by disease stage and host risk, while emphasizing key translational gaps, particularly validation in mixed-species biofilms and long-term biocompatibility.

## 1 Introduction

Dental implant longevity is increasingly threatened by biofilm-associated infections, as conventional therapies often fail against bacterial resistance and the extracellular polymeric substance (EPS) barrier (1–5). Nanotechnology offers a paradigm shift via two primary strategies: (1) Surface functionalization for passive defense (6, 7); (2) Nanocarrier-based systems for active, targeted eradication (8, 9). This mini-review synthesizes frontier nanostrategies, elucidating mechanisms facilitating dual “antimicrobial-osteogenic” efficacy—such as immunomodulation and physical puncture (10, 11), while assessing critical translational barriers regarding biosafety and scalability (12, 13).

**Figure 1:**
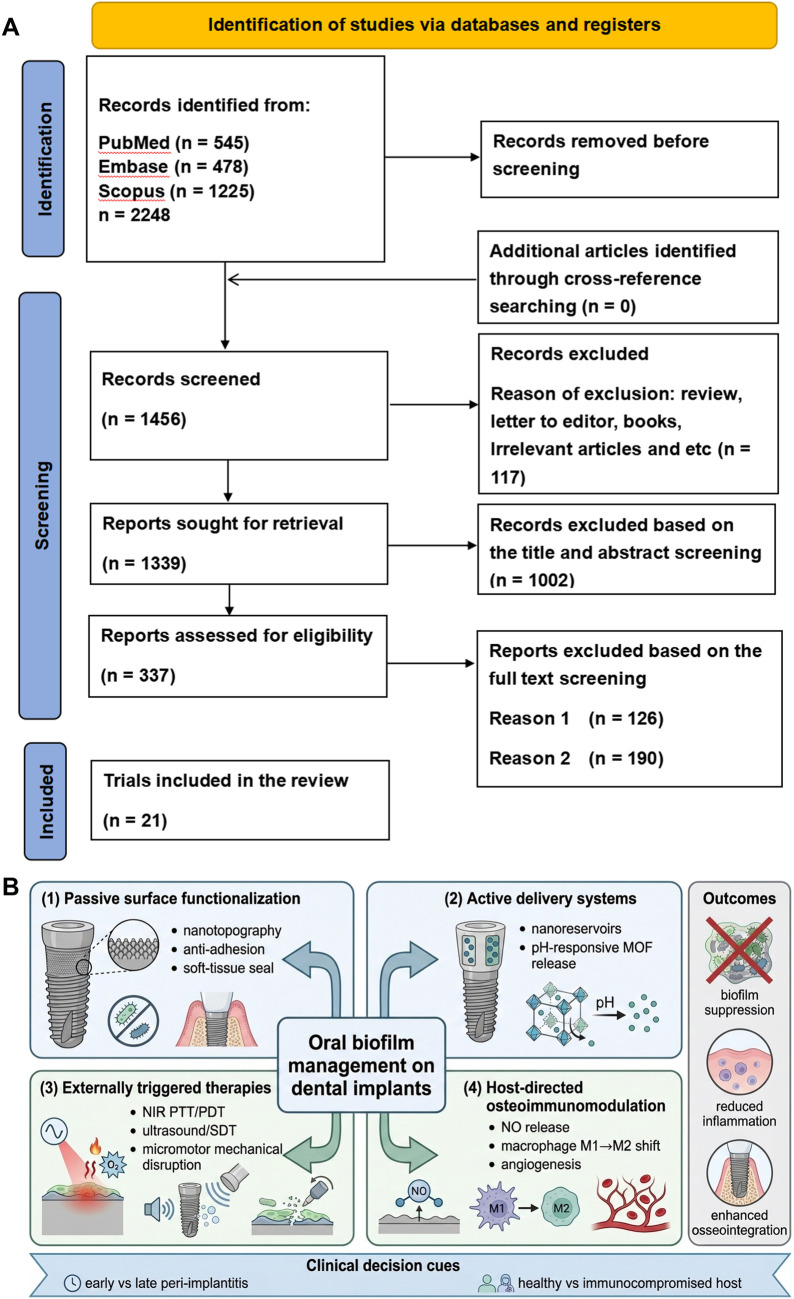
**(A)** The PRISMA flow diagram. **(B)** An illustration of the classification of the four nanomaterial-based strategies.

A targeted literature search was conducted to support this narrative mini-review. PubMed, Embase, and Scopus were searched for English-language studies published between January 2015 and January 2025, with the final search updated on 10 February 2026. A combination of controlled vocabulary and free-text terms was used, including peri-implantitis/peri-implant disease, oral biofilm, implant infection, and nanomaterials. An example search string for PubMed was: (“peri-implantitis” OR “peri-implant disease”) AND (“biofilm” OR “oral biofilm”) AND (“nanomaterial*” OR “nanoparticle*” OR “nanotopograph*” OR “drug delivery” OR “photothermal” OR “photodynamic” OR “metal-organic framework”). Studies were selected for narrative synthesis when they were peer-reviewed, implant-related, and reported explicit anti-biofilm, antimicrobial, or peri-implant tissue-response outcomes. Representative strategies are summarized in Table 1, and a PRISMA flow diagram together with the overall classification of the strategies is illustrated in Figure 1.

**Table 1:** Integrated overview of nanomaterial-based strategies for infection control, biocompatibility, and generalized modes of action.

Strategy Category	Nanomaterial and Fabrication Method	Evidence Level	Antibacterial Efficacy and Target Pathogens	Biocompatibility and Histological Activity	Core Mechanism of Action
Passive Defense	Disordered TiO2 nanoneedles via hydrothermal synthesis (5)	Disordered TiO₂ nanoneedles via hydrothermal synthesis (5/7 in current text) *in vitro* + *in vivo* (small-animal/preclinical)	Ruptured membranes of *S. aureus* and E. *coli* through physical contact stress, achieving over 90 percent killing efficiency.	Upregulated FAK expression and promoted osteoblast spreading and filopodia extension.	Mechano-bactericidal Topography: High-aspect-ratio nanoneedles impose tensile/contact stress that physically ruptures bacterial membranes, killing bacteria without chemical agents.
Passive Defense	Additively manufactured Ti-6Al-4 V via selective laser melting (22)	Additively manufactured Ti-6Al-4 V via selective laser melting (22/26 depending on renumbering) Preclinical engineering study/*in vitro*	Surface roughness of approximately 24 micrometers inhibited the adhesion of *S. aureus* and *P. aeruginosa*.	Enhanced mechanical interlocking and stability, suitable for rapid pediatric rehabilitation.	Mechano-bactericidal Topography/Physical anti-adhesion: Micro-rough topography creates an unfavorable interface for initial bacterial attachment, reducing adhesion and subsequent biofilm initiation.
Passive Defense	Ultra-dense polymer brushes via surface-initiated ATRP (2)	Ultra-dense polymer brushes via surface-initiated ATRP (2) *in vitro* + *in vivo*	Reduced accumulation of *S. epidermidis* by more than 3 orders of magnitude and prevented infection *in vivo*.	Demonstrated excellent biocompatibility with no fibrous capsule formation or inflammatory response.	Anti-fouling and Steric Repulsion: Hydrophilic polymer brushes/hydration layers form a physical and steric barrier that repels protein adsorption and prevents initial bacterial attachment.
Passive Defense	Peptide-functionalized titanium via silanization (17)	Peptide-functionalized titanium via silanization (17/18 depending on renumbering) *in vivo* (soft-tissue integration model)	Prevented apical migration of oral bacteria by establishing a biological seal at the soft tissue interface.	Enhanced hemidesmosome formation in gingival epithelial cells, promoting soft tissue integration.	Bio-mimetic Soft Tissue Seal: Bioactive peptides/proteins promote rapid gingival epithelial adhesion to form a biological barrier that blocks bacterial ingress at the peri-implant interface.
Passive Defense	Internal silver nanoparticle coating via immersion (10)	Internal silver nanoparticle coating via immersion (10/12 depending on renumbering) *in vitro*/*ex vivo* contamination model	Eliminated leakage of *C. albicans* and *E. faecalis* from the implant-abutment interface gap.	Preserved marginal bone levels by removing internal inflammatory stimuli.	Anti-fouling/Interface infection blocking (gap control): Antimicrobial coating confined to the implant–abutment microgap suppresses colonization and prevents microbial leakage from the internal interface.
Active Delivery	pH-responsive MOF (ZIF-8) via hydrothermal synthesis (18)	pH-responsive MOF (ZIF-8) via hydrothermal synthesis (18/21 depending on renumbering) *in vitro* + *in vivo* (preclinical)	Released Zinc ions and imidazole only at pH 5.5, effectively killing bacteria in acidic environments.	Released Zinc ions upregulated osteogenic genes ALP and Runx2, enhancing PEEK osseointegration.	Microenvironment-responsive Release: MOF carriers stay stable at physiological pH but disintegrate under infection-associated acidity, triggering on-demand release of antimicrobial species.
Active Delivery	Sandwich-type degradable coating via layer-by-layer assembly (4)	Sandwich-type degradable coating via layer-by-layer assembly (4/6 depending on renumbering) *in vitro* + *in vivo* (small-animal/preclinical)	Outer layer released antimicrobials to prevent acute peri-operative infection.	Inner layer degradation exposed osteoconductive cues, inducing macrophage M2 polarization.	Sequential Functionalization: Layer-by-layer “time-programmed” coatings first release antimicrobials for acute infection control, then expose osteoconductive/host-regenerative cues during remodeling; Osteoimmunomodulation (secondary): surface cues bias macrophages toward a pro-healing M2 phenotype.
Active Delivery	Drug-loaded TiO2 nanotubes via anodization (16, 20)	Drug-loaded TiO₂ nanotubes via anodization (16, 20/16,23 depending on renumbering) *in vitro* + *in vivo*	Sustained release of BSA or Gentamicin significantly inhibited *S. aureus* growth post-operation.	Nanotubular topography enhanced osteoblast adhesion and minimized systemic toxicity.	Sustained Local Release (depot effect): TiO2 nanotubes act as local drug reservoirs enabling sustained release to inhibit postoperative bacterial growth while limiting systemic exposure.
Active Delivery	Synergistic loading of Silver and Antibiotics on Graphene Oxide (14, 15)	Synergistic loading of Silver and Antibiotics on Graphene Oxide (14, 15/13,16 etc.) Primarily *in vitro*; partial *in vivo* support	Silver and Graphene Oxide destabilized cell walls, enhancing antibiotic permeability against resistant strains.	Synergistic effect allowed for lower drug dosages, preserving osteoblast viability and proliferation.	Synergistic Permeabilization: Nanomaterials destabilize bacterial cell walls/membranes, increasing permeability and intracellular accumulation of co-delivered antibiotics to improve efficacy against resistant strains.
Active Delivery	Porous beta-titanium with biopolymer/silver coating via 3D printing (6)	Porous beta-titanium with biopolymer/silver coating via 3D printing (6/8) *in vitro* + *in vivo* (preclinical)	Demonstrated potent broad-spectrum activity against both *S. aureus* bacteria and *C. albicans* fungi.	Low elastic modulus reduced stress shielding, while porous structure facilitated bone ingrowth.	Microenvironment-responsive/contact-active chemical therapy (silver-based) with structural support: Silver-containing coatings provide broad-spectrum antimicrobial action, while the porous, low-modulus scaffold supports tissue ingrowth and reduces stress shielding (structure–function coupled infection control).
External Field	Photothermal agents (Gold nanostars, Black Phosphorus) (19, 21)	Photothermal agents (Gold nanostars, Black Phosphorus) (19, 21/22,24) *in vitro* + *in vivo* (preclinical)	Near-infrared irradiation generated heat over 50 degrees Celsius to rapidly eradicate *S. aureus* biofilms.	Nanostructure and Strontium release promoted osteogenesis via mechanotransduction pathways.	Photothermal Therapy: NIR absorption by photothermal agents generates localized hyperthermia that ablates biofilms and denatures bacterial proteins with spatiotemporal control.
External Field	ROS generators (Piezoelectric, Upconversion) (3, 13)	ROS generators (Piezoelectric, Upconversion) (3, 13/3,25) *in vitro* + *in vivo* (preclinical)	Ultrasound or light excitation generated reactive oxygen species to penetrate deep into biofilms.	Modulated macrophage polarization towards the regenerative M2 phenotype and minimized thermal damage.	Sonodynamic and Photodynamic Therapy (ROS-mediated): Ultrasound/light activates sensitizers to generate cytotoxic ROS (e.g., singlet oxygen, hydroxyl radicals) that damage bacterial DNA and lipids, including within biofilms.
External Field	Janus micromotors via self-assembly (1)	Janus micromotors via self-assembly (1) *in vitro* proof-of-concept	Active propulsion driven by oxygen bubbles mechanically scrubbed and dislodged *P. gingivalis* biofilms.	Oxygen generation alleviated local hypoxia, favoring tissue healing and fibroblast survival.	Active Physical Disruption: Self-propelled micromotors generate kinetic force/motion (e.g., bubble propulsion) to physically scrub and detach established biofilms from implant surfaces.
Host Modulation	Biomimetic multifaceted titanium via micro-nano texturing (9)	Biomimetic multifaceted titanium via micro-nano texturing (9/11) *in vitro* + *in vivo* (preclinical)	Hierarchical topography significantly reduced the available surface area for bacterial adhesion.	Modulated the immune microenvironment towards an anti-inflammatory M2 state.	Osteoimmunomodulation: Surface chemical/physical cues regulate macrophage polarization toward a regenerative M2 phenotype, creating a pro-healing microenvironment that lowers infection susceptibility; Anti-adhesion (secondary): hierarchical textures reduce effective adhesion sites.
Host Modulation	Multifunctional CFR-PEEK via composite molding (8)	Multifunctional CFR-PEEK via composite molding (8/10) *in vitro* + *in vivo* (preclinical)	Rapid eradication of bacteria via photothermal and photodynamic therapy under light irradiation.	Promoted the coupling of angiogenesis and osteogenesis via VEGF upregulation.	Angio-osteogenic Coupling: Material cues enhance endothelial–osteoblast crosstalk so vascularization and osteogenesis proceed synchronously for functional osseointegration; Photothermal + Photodynamic Therapy (secondary): light-triggered heat/ROS contribute to rapid bacterial eradication.
Multimodal	Integrated multimodal systems (7, 11)	Integrated multimodal systems (7, 11/9,13 etc.) *in vitro* + *in vivo* (preclinical)	Combined thermal, oxidative, and chemical therapies achieved complete eradication of established biofilms.	Supported fibroblast and osteoblast attachment while preventing the development of bacterial resistance.	Integrated Multimodal Systems: Combines multiple bactericidal modes (thermal, oxidative/ROS, chemical) to eradicate established biofilms and reduce resistance risk while maintaining tissue compatibility.

TiO2, titanium dioxide; Ti-6Al-4 V, titanium–6 aluminum–4 vanadium alloy; ATRP, atom transfer radical polymerization; FAK, focal adhesion kinase; MOF, metal–organic framework; ZIF-8, zeolitic imidazolate framework-8; PEEK, polyetheretherketone; ALP, alkaline phosphatase; Runx2, runt-related transcription factor 2; BSA, bovine serum albumin; ROS, reactive oxygen species; NIR, near-infrared; CFR-PEEK, carbon fiber-reinforced polyetheretherketone; VEGF, vascular endothelial growth factor; *S. aureus*, *Staphylococcus aureus*; *E. coli*, *Escherichia coli*; *S. epidermidis*, *Staphylococcus epidermidis*; *P. aeruginosa*, *Pseudomonas aeruginosa*; *C. albicans*, *Candida albicans*; *E. faecalis*, *Enterococcus faecalis*; *P. gingivalis*, *Porphyromonas gingivalis*.

The “Core Mechanism of Action” column integrates the conceptual mechanism categories originally summarized in former Table 2; therefore, former Table 2 has been removed to avoid duplication.

“Secondary” indicates an auxiliary mechanism that contributes to the observed biological effect but is not the principal defining mechanism of the platform.

Recent complementary references relevant to polymicrobial/fungal biofilms, long-term biosafety, drug delivery, and clinical translation include Biomaterials science and surface engineering strategies for dental peri-implantitis management (2024), Cross-kingdom microbial interactions in dental implant-related infections: is *Candida albicans* a new villain? (2022), The current applications of nano and biomaterials in drug delivery of dental implant (2024), Future prospects in clinical translation of inorganic in medical devices using nanomaterials and nanotechnology: Innovation and regulatory science (2025), and Nanotechnology in healthcare, and its safety and environmental risks (2024).

## 2 Surface topography and passive defense mechanisms

### 2.1 Nanostructural engineering and mechanical bactericidal effects

Disordered high-aspect-ratio nanoneedle arrays on titanium surfaces can rupture microbial membranes through local mechanical stress, thereby producing agent-free, broad-spectrum contact-killing activity against mixed-species biofilms, including both bacteria and fungi (7). Similarly, nanostructured topographies that rely on a mechanical bactericidal mechanism can physically penetrate cell walls, thereby exerting non-specific, broad-spectrum antimicrobial activity against complex mixed-species biofilms. Unlike antibiotics that target specific molecular sites, such physical surface features can overcome the limitations inherent in strategies designed for a single microbial species, and have demonstrated significant contact-killing efficacy against both bacteria and fungi (7). Grain refinement strategies, such as ultrafine-grained titanium coated with graphene oxide (GO), enhance antimicrobial performance by altering surface energy and increasing grain boundary density (14). GO nanosheets act as “nanoknives” that damage bacterial membranes, while planar modifications mainly rely on tangential shear forces and thermodynamic repulsion (15). Although TiO2 nanotubes can influence cellular behavior and also serve as drug reservoirs (15, 16), their intrinsic bactericidal activity is generally weaker than that of high-curvature nanoneedles. However, conditioning layer formation, including necrotic bacterial debris and adsorbed host proteins, may obscure sharp nanostructures and reduce their long-term mechanical bactericidal effect (7, 14). Consequently, it is essential to develop surfaces that combine physical bactericidal action with self-cleaning or degradation capabilities to maintain long-term biological activity.

However, the mechanistic basis of “mechanical killing” should be interpreted cautiously. Current evidence supports both direct contact-induced rupture and indirect stress-mediated membrane instability, and these two explanations are not necessarily mutually exclusive. In practice, the *in vivo* situation is further complicated by conditioning films composed of salivary proteins, host macromolecules, and microbial debris, which may mask sharp nanofeatures, alter wettability, and reduce effective bacteria–surface contact. Accordingly, long-term durability should be evaluated under saliva-relevant protein adsorption and repeated exposure conditions rather than inferred solely from static imaging or short-term mono-species assays.

### 2.2 Anti-adhesion chemistry and biomimetic soft tissue integration

Polymer brush coatings reduce initial bacterial adhesion by forming dense hydration layers that create steric repulsion, although they cannot actively eliminate bacteria that cross this barrier (2). By contrast, basement membrane-inspired modifications, particularly laminin-derived peptides, enhance epithelial attachment and promote a soft-tissue seal that blocks peri-implant pathogen ingress (17). This barrier-oriented strategy favors rapid host tissue integration while reducing reliance on direct bactericidal activity. Oral biofilms are typically complex communities composed of both bacteria and fungi, such as Candida albicans (18, 19). In such cross-kingdom interactions, physical defense strategies demonstrate broader applicability than species-specific antibiotics. Mimicking the soft-tissue sealing interface of the natural gingiva can establish a robust physical barrier that must not only prevent bacterial colonization, but also resist fungal adhesion and invasion, which is particularly important in immunocompromised hosts (17).

## 3 Functional coatings for controlled delivery and responsiveness

### 3.1 Nanoreservoirs and inorganic antimicrobial agents

Nanostructured surfaces are less effective against established or deep-seated biofilms, making nanoreservoirs with high loading capacity and controlled release important for sustained antimicrobial activity. TiO2 nanotube arrays on titanium are widely used as antibiotic carriers because their tunable tubular architecture enables efficient drug loading (15, 16). Antibiotic-loaded nanotubes reduce Staphylococcus aureus viability and can enhance other antimicrobial modalities through synergistic effects (15, 16). For bioinert substrates such as polyetheretherketone (PEEK), MOF-based core–shell structures provide an alternative platform for loading hydrophobic drugs and compensating for limited surface bioactivity (20).

Inorganic metal ions are attractive antimicrobial agents because they provide broad-spectrum activity with a relatively low risk of resistance. Silver nanoparticles (AgNPs) disrupt bacterial membrane potential and enzymatic function through the release of silver ions, while green synthesis can help reduce host cytotoxicity (13). This broad-spectrum antimicrobial activity offers a significant advantage in the management of complex mixed-species biofilms involving bacterial–fungal interactions (18, 19). A purely antibacterial approach may result in fungal overgrowth, whereas the incorporation of broad-spectrum agents such as silver nanoparticles (AgNPs) can simultaneously suppress both microbial groups. For example, silver nanoparticles not only exert bactericidal effects, but also effectively reduce Candida albicans contamination within implant structures, thereby providing a dual antimicrobial effect against polymicrobial communities (12). Loading silver nanoparticles into biopolymer coatings or suspensions has been proven effective in inhibiting fungal colonization, including *Candida albicans*, thereby expanding the antimicrobial spectrum (8, 13). Cross-kingdom interactions may increase peri-implant biofilm resilience by stabilizing matrix architecture, altering microbial spatial organization, and reducing the apparent efficacy of purely antibacterial approaches. This is particularly relevant for Candida-associated mixed biofilms, in which antifungal coverage becomes an important complement to antibacterial activity. Therefore, broad-spectrum nanomaterials such as silver-based systems may be advantageous not only because they suppress bacteria, but also because they can reduce fungal contamination within implant-associated niches. Future evaluation frameworks should more consistently include mixed bacterial–fungal biofilms when reporting antimicrobial performance.To balance antimicrobial activity with osteogenicity, osteoinductive elements such as strontium (Sr) are often introduced to construct dual-ion systems. For instance, the co-modification of zirconia (ZrO2) surfaces with strontium and two-dimensional black phosphorus nanosheets not only utilizes the photothermal effect of black phosphorus but also promotes osseointegration through the release of strontium ions (21). Studies using BSA as a model molecule have further validated the universal loading and release capabilities of nanotube-based delivery systems for macromolecular biologics (22).

### 3.2 Microenvironment-responsive and sequential release systems

Traditional coatings may cause burst release, local cytotoxicity, and later subtherapeutic drug levels. To address this, pH-responsive MOF coatings remain stable under physiological conditions but release drugs in acidic infection-associated environments, thereby enabling on-demand antimicrobial delivery (20).

Sequential-release systems are designed to match the timeline of tissue repair by delivering bactericides early and immunomodulatory or osteogenic signals later (6). This staged strategy aligns with the clinical sequence of infection control followed by tissue regeneration and may also improve the peri-implant immune microenvironment through macrophage polarization (6).

## 4 Active therapies triggered by external fields

### 4.1 Photothermal and photodynamic synergistic effects

Traditional passive coatings often fail to penetrate the dense extracellular polymeric substance (EPS) matrix of mature biofilms. Utilizing the localized surface plasmon resonance (LSPR) effect under near-infrared (NIR) light, gold nanostars have been confirmed to rapidly convert NIR energy into localized hyperthermia, causing bacterial membrane rupture and protein denaturation while promoting osteogenesis through thermal stimulation (23). Two-dimensional black phosphorus (BP) nanosheets integrated on zirconia implants exhibit extremely high photothermal conversion efficiency due to their tunable bandgap, and their degradation products (phosphate groups) serve as mineralization precursors to enhance osseointegration (21). The characteristics of deep tissue penetration and minimal background absorption make these materials widely used for triggering photothermal therapy (PTT) and photodynamic therapy (PDT) on implant surfaces.

Single-mode therapies are often limited by the thermal tolerance of surrounding tissues or the extremely short lifespan of reactive oxygen species (ROS). In synergistic strategies combining PTT with chemical or gas therapy, polydopamine (PDA) coatings functionalized with NO donors demonstrate that mild photothermal heating increases bacterial membrane permeability, facilitating the deep penetration of therapeutic gases like nitric oxide (NO) into the biofilm (9). This “thermal-assisted permeation” mechanism significantly enhances biofilm clearance efficiency, with the released NO simultaneously promoting angiogenesis and immunomodulation (9). Combining PTT with ROS-generating PDT also achieves remote clearance of established *in vivo* biofilms by utilizing high temperatures to sensitize bacteria to oxidative stress (3).

### 4.2 Sonochemistry and mechanical disruption

Ultrasound (US) offers greater penetration depth than light and avoids the risk of thermal damage, making it suitable for treating deep-seated infections. Piezoelectric hybrid coatings exploit sonochemical effects to catalyze ROS generation under ultrasound irradiation (24). The polarization of piezoelectric materials facilitates the separation of electron-hole pairs, driving free radical chain reactions that degrade the organic biofilm matrix and induce macrophage polarization towards an anti-inflammatory phenotype (24).

Active physical disruption provides a means to dismantle biofilm structures. utilizing the catalytic decomposition of hydrogen peroxide within the micropores of diatomite, self-powered microbubble generators create recoil forces that drive the autonomous motion of micromotors (1). The mechanical shear forces generated by this active motion physically scrub and disrupt the three-dimensional structure of the biofilm, exposing protected bacteria to antimicrobial agents and overcoming the limitations of static diffusion therapies (1).

Despite these promising results, the evidence summarized in this section remains predominantly preclinical. Most externally triggered platforms and multifunctional coatings discussed here have been validated *in vitro* and/or in small-animal models, whereas large-animal validation is uncommon and human clinical evidence is not represented among the representative studies summarized in this mini-review. Accordingly, apparent efficacy should not be interpreted as near-clinical readiness, and translational claims must remain cautious.

## 5 Immunomodulation and osteo-angiogenic coupling

### 5.1 Macrophage polarization and immune microenvironment remodeling

The concept of “osteoimmunomodulation” emphasizes the simultaneous achievement of anti-infection and osseointegration by regulating the immune microenvironment. Biomimetic coatings on titanium surfaces can modulate the transition of macrophages from the pro-inflammatory M1 phenotype to the anti-inflammatory/reparative M2 phenotype (11). This immunomodulatory property not only mitigates excessive inflammatory responses but also indirectly promotes osteoblast differentiation through the secretion of cytokines such as BMP-2 and VEGF (11). TiO2 surfaces with disordered nanoneedle structures have been confirmed to regulate the osteoimmune microenvironment, inhibiting osteoclastogenesis while enhancing osteogenic activity alongside bacterial killing (7).

For the distinct phases of infection and regeneration, degradable triple-layer sandwich coating designs achieve temporal regulation of the immune response: during the acute infection phase, rapidly released bactericides assist M1 macrophages in clearing pathogens; as the coating degrades, subsequently exposed bioactive layers induce M2 polarization, thereby initiating tissue repair and remodeling (6). This staged strategy significantly improves implantation success rates by matching the host's natural healing cascade (6).

### 5.2 Osteo-Angiogenic coupling and multifunctional synergy

Angiogenesis is closely coupled with and mutually reinforces osteogenesis. Functional modification of carbon fiber-reinforced polyetheretherketone (CFR-PEEK) implants via the introduction of photothermal and bioactive nanocoatings can achieve rapid bacterial clearance, upregulate vascular endothelial growth factor (VEGF) expression, promote the formation of neovascular networks, and accelerate deep osseointegration (10).

Nitric oxide (NO), as a critical gas signaling molecule, has been confirmed to possess triple functions: antibacterial activity, angiogenesis, and immunomodulation. NIR-triggered NO release systems not only synergistically disrupt biofilms but also improve local blood supply through vasodilation and modulate inflammatory responses to favor bone repair (9). Addressing the rapid bone healing needs of special populations such as children, customized porous titanium scaffolds combined with surface modifications using 3D printing technology can achieve highly efficient sterilization and osseointegration within a short timeframe (25).

## 6 From benchtop complexity to clinical viability

### 6.1 Mechanism elucidation and evaluation model innovation under biological complexity

While nanotopography and ROS have validated non-antibiotic control of biofilm-associated infections, current reliance on simplified single-species models still underestimates the polymicrobial and occasional cross-kingdom complexity of peri-implant biofilms (18, 19). Future models should therefore more routinely incorporate mixed-species conditions, including fungal participation where relevant (12, 18, 19, 26).

As discussed in Section 2.1, current evidence supports both direct contact-induced rupture and indirect stress-mediated membrane instability as contributors to nanotopography-mediated killing. The key remaining gap is not simply which mechanism is plausible, but how these effects behave under saliva-conditioned, protein-coated, and polymicrobial conditions over time. Future work should therefore combine force-based measurements, live imaging, and saliva-relevant exposure models to quantify durability and loss of activity under clinically relevant conditions.

The *in vivo* safety threshold for therapeutic agents, particularly reactive oxygen species (ROS), has yet to be clearly established (27, 28). Although photodynamic and sonodynamic therapies have been shown to effectively eradicate persistent biofilms, there remains a lack of systematic investigation in complex *in vivo* environments regarding the diffusion radius of ROS and the boundaries of oxidative damage inflicted on surrounding stem cells or vascular endothelial cells. Most existing studies report only the antibacterial and osteogenic effects achieved under “optimal parameters,” while rarely defining the upper limit of ROS concentrations that can be tolerated by host tissues (7, 12), highlighting the need for dose–response analysis. In contrast, non-ROS-based physical strategies, such as nanospikes or anti-adhesive polymers (8, 11), can avoid the risks associated with chemical or oxidative injury. Therefore, future research should not remain limited to reporting therapeutic efficacy alone, but must instead quantify the therapeutic window of ROS and, in physiologically relevant three-dimensional co-culture models, systematically compare the advantages and limitations of active oxidative attack vs. passive physical defense at different stages of infection, in order to determine the optimal clinical intervention strategy.

### 6.2 Synergistic optimization of physicochemical stability and tribological performance

Long-term evidence regarding the stability of functional coatings and the metabolic fate of degradation products remains insufficient (27–29). The designs of triple-layer degradation coatings (6) and black phosphorus nanosheets (21) achieve sequential functional release, offering insights for infection control during early osseointegration. However, during the bone remodeling period, which spans months or even years, robust long-term histological and biodistribution data remain limited regarding whether degradation products (e.g., silver ions, phosphate groups, polymer fragments) accumulate in local lymphatic tissues or distant organs such as the liver, spleen, and kidneys (27, 29). For dental implants intended for long-term retention, this absence of long-term biocompatibility data is critical. Beyond ROS-related oxidative injury, biosafety assessment should also address ion-specific toxicity, systemic biodistribution, and ecological effects on the oral microbiome. For example, silver-, zinc-, or phosphate-releasing systems may achieve useful antimicrobial activity, but their long-term local concentration ranges, cumulative tissue exposure, and organ-level distribution remain insufficiently defined. In addition, broad-spectrum antimicrobial strategies may unintentionally disturb commensal oral microbial communities, which could alter recolonization dynamics after treatment. Accordingly, future biosafety evaluation should integrate local cytocompatibility, long-term biodistribution, and microbiome-level analyses rather than focusing on single-parameter toxicity alone. Antibiotic-loaded nanotubes (16) or polymer brushes (2) face the risk of delamination due to high shear forces during implantation surgery; delamination not only leads to functional failure but may also induce aseptic inflammation. Therefore, material development will gradually shift from simple surface deposition to integral modification of the matrix and surface. Utilizing severe plastic deformation (SPD) to fabricate ultrafine-grained materials (14) or employing 3D printing for pore size/porosity gradient design in porous structures (25) can endow materials with endogenous antimicrobial or osseointegration capabilities without relying on exogenous coatings, thereby fundamentally avoiding the risk of coating delamination.

Dental implant biotribology should be evaluated under clinically relevant cyclic masticatory loading rather than inferred from static material properties alone. In the oral cavity, repeated occlusal forces, micromotion at implant-associated interfaces, saliva-mediated lubrication, and stress concentration around porous or coated structures may together accelerate fretting wear, material fatigue, and local particle release (8). These processes are highly relevant to peri-implant bone stability because they may contribute to osteolysis, interface weakening, or loss of long-term functional integrity. Therefore, future material design should integrate dental-specific biotribological testing under saliva-relevant and mastication-mimicking conditions (30).

### 6.3 Feasibility assessment and regulatory adaptability for clinical translation

Current microenvironment-responsive systems, such as pH-sensitive MOF coatings, can respond to acidic infection environments but generally lack the ability to distinguish specific pathogens. As a result, they may also affect commensal oral flora (5). The development of therapeutic specificity and intelligence lies in integrating specific enzyme substrates or quorum sensing (QS) inhibitors to achieve “targeted eradication” solely against key pathogens such as *Porphyromonas gingivalis*.

External field-responsive therapies [e.g., NIR photothermal (9, 23)] are potent but require multiple patient visits. Future development should prioritize portable, ergonomically designed photoacoustic or acoustic-excitation devices that improve access to periodontal pockets or peri-implant bone defects, thereby reducing reliance on professional medical resources and improving patient compliance.

The selection of nanotechnology-based strategies may also vary according to the stage of disease progression. For early peri-implant mucositis, passive defense approaches primarily based on anti-adhesive coatings or mechanically bactericidal nanotopographies may be sufficient to prevent biofilm formation (2, 7, 8). By contrast, once biofilms are established or overt infection is present, passive strategies alone may be insufficient and may need to be combined with physical field activation or stimuli-responsive drug delivery systems to achieve more effective antimicrobial treatment (3, 9, 20, 21, 23, 24).

With regard to patient-related factors, in individuals with certain systemic conditions, such as osteoporosis or immunodeficiency, coatings that integrate osteoimmunomodulatory and antibacterial functions can not only control infection, but also reconstruct an immune microenvironment conducive to tissue repair (6, 11, 24).

From a regulatory perspective, strategies involving the delivery of novel agents, such as gaseous molecules or complex synthetic materials, are subject to stringent scrutiny (1, 20, 28, 31). Accordingly, research may shift toward approaches such as surface topography design, biomimetic soft-tissue sealing modifications, or nanomodification using widely applied agents, such as silver and tetracycline (7, 8, 12–14, 17). These strategies, which do not involve the release of novel therapeutics or instead rely on clinically well-established agents, are more likely to obtain regulatory approval and thereby accelerate clinical translation.

Customization for specific populations and regulatory adaptation remain important for clinical translation. Given the limited systemic safety data for long-term implanted nanomaterials, short-term implant applications may offer a more feasible translational pathway; for example, additively manufactured implants for pediatric fracture fixation (25) represent a removable application scenario that may reduce long-term toxicity concerns. From a regulatory perspective, drug–device combination products involving gas signaling molecules or drug loading are likely to face stricter scrutiny, whereas purely physical or biomimetic strategies, such as surface topography design (7) or basement membrane peptide modification (17), may be more readily translated because they do not rely on exogenous drug release. In parallel, scalable and greener manufacturing routes will be essential, as many high-performing nanomaterials still face challenges related to batch-to-batch consistency and cost (13).

## 7 Conclusion

Nanomaterial-based strategies have catalyzed a paradigm shift in the management of peri-implantitis, transitioning from passive anti-biofilm coatings to dynamic, multi-functional systems. By integrating external field responsiveness with osteoimmunomodulation, these advanced platforms offer a potent solution to the critical “infection-integration” paradox.

Importantly, evidence maturity differs substantially across the strategies discussed in this mini-review. Most nanotopographical surfaces, responsive coatings, externally triggered systems, and multifunctional platforms remain supported predominantly by *in vitro* studies and small-animal/preclinical models. Large-animal validation is uncommon, and human clinical evidence is not represented among the representative studies summarized here. Therefore, promising antimicrobial or osteogenic outcomes should be interpreted as preclinical signals rather than indicators of near-clinical readiness.

From a translational perspective, simpler platforms such as anti-adhesive coatings, soft-tissue sealing interfaces, or mechanically active surfaces may be attractive because of their lower design complexity, whereas externally triggered and multifunctional systems may provide stronger biofilm eradication but introduce additional constraints related to device dependence, exposure control, and long-term safety. Future progress will require standardized evidence pipelines, saliva-relevant durability testing, and clinically relevant polymicrobial models before genuine translational readiness can be claimed.

Given the limited long-term safety and efficacy data for nano-modified implantable devices, future studies should move beyond short-term, publication-driven designs and prioritize validation in complex host models that better reflect clinical reality, particularly those involving polymicrobial communities and immunocompromised conditions (12). At the same time, successful translation will depend on addressing persistent challenges related to biosafety, tribological stability, and scalable manufacturing.
